# Targeting endothelial cells: the pathological mechanisms and therapeutic innovations in pulmonary arterial hypertension

**DOI:** 10.3389/fcell.2025.1690124

**Published:** 2025-11-26

**Authors:** Tianfei Fan, Longji Li, Yao Wang, Meng Lin, Fengbo Wu

**Affiliations:** 1 Department of Pharmacy, West China Hospital, Sichuan University, Chengdu, China; 2 Strength and Conditioning Training Center, School of Physical Education, Chengdu Sport University, Chengdu, China

**Keywords:** pulmonary arterial hypertension, endothelial cells, endothelial-mesenchymal transition, crosstalk, stem cell treatment

## Abstract

Pulmonary arterial hypertension (PAH) is a fatal disease with high mortality, which is characterized by pulmonary vascular remodeling. Current clinical treatments mainly focus on dilating vascular and relieving pulmonary artery pressure. However, there is still no effective treatment available to reverse vascular remodeling. Endothelial cells (ECs) play an important role in vascular function and repair. Endothelial dysfunction is a key factor inducing vascular remodeling and PAH. The endothelial - mesenchymal transition (EndMT), along with the abnormal apoptosis and proliferation of endothelial cells (ECs) disrupt vascular homeostasis and drive pulmonary artery remodeling. These regulated by the TGF - β/BMP, PI3K/Akt, and JAK - STAT pathways. Moreover, the secretion of active factors by ECs and the crosstalk between ECs and smooth muscle cells (SMCs) also influence vascular remodeling. Targeting ECs shows certain application prospects in the treatment and diagnosis of PAH. This article elaborates on the role and mechanisms of ECs in PAH, and reviews their diagnostic markers and therapeutic targets for the early intervention and effective management of PAH.

## 1 Introduction

Pulmonary arterial hypertension (PAH) is a complex and progressive pulmonary disease associated with high mortality (Johnson et al., 2023). The incidence of PAH among adults is 47.6–54.7 per million people. The annual incidence of PAH is 5.8% and it is on the rise (Thenappan et al., 2018a). The main pathological features of PAH are persistent vasoconstriction and pulmonary vascular remodeling. Endothelial dysfunction serves as an initiating factor for vascular remodeling. Endothelial dysfunction is defined as a state in which endothelial cells lose their normal physiological functions, including regulation of vascular tone, barrier integrity, anticoagulant properties, and angiogenic capacity, leading to vascular imbalance and pathological remodeling (Correale et al., 2025). It is characterized by the abnormal proliferation and apoptosis of pulmonary arterial endothelial cells (PAECs), as well as endothelial - mesenchymal transition (EndMT) (Rajagopal and Yu, 2022). These processes cause vascular thickening and hardening, which ultimately lead to right heart failure (Shah et al., 2022).

Endothelial cells (ECs) are stable single - layer squamous epithelial cells lining the vascular lumen. They mainly facilitate the metabolic exchange between plasma and interstitial fluid. ECs synthesize and secrete bioactive factors to maintain vascular tone and internal homeostasis (Hennigs et al., 2021). In PAH, ECs produce vasoconstrictors, such as endothelin - 1 (ET - 1) and thromboxane, as well as vascular endothelial growth factor (VEGF) (Evans et al., 2021). This production leads to a reduction in the expression of nitric oxide synthase and prostacyclin synthase, resulting in ECs proliferation, apoptosis, endothelial dysfunction, abnormal angiogenesis, and thrombosis (Wang and Valdez-Jasso, 2021). The abnormal activation of ECs causes various diseases, including inflammation, cardiovascular disease, and pulmonary vascular disease.

Currently, the approved treatments for PAH mainly target three signaling pathways: nitric oxide (NO), prostacyclin (PGI2), and endothelin-1 (ET-1). The therapeutic drugs are NO donors, PGI2 receptor agonists, and ET - 1 receptor antagonists (Christou and Khalil, 2022). However, these drugs primarily promote vasodilation and fail to reverse vascular remodeling, thereby limiting their therapeutic efficacy. Although animal models, such as hypoxia or monocrotaline induced PAH in rodents, have provided valuable insights into disease mechanisms, they cannot fully recapitulate the complexity of human PAH, including genetic heterogeneity, comorbidities, and long-term disease progression. Consequently, there is an urgent need to develop novel therapeutic strategies and targets for PAH. This review focuses on the role of ECs in PAH pathogenesis and explores the potential of targeting ECs as a novel approach for PAH diagnosis and treatment.

## 2 Methods

The references included in this mini review were retrieved from PubMed, Web of Science, Scopus, and Embase databases. The search covered the period from January 2015 to November 2025. Medical subject headings (MeSHs) were used and categorized into three thematic groups: (1) PAH-related terms (“pulmonary arterial hypertension,” “PAH,” or “pulmonary vascular remodeling”); (2) endothelial cell-related terms (“endothelial cells,” “pulmonary arterial endothelial cells,” “PAECs,” or “endothelial-mesenchymal transition,” “EndMT”); and (3) mechanistic/therapeutic terms (“TGF-β/BMP signaling,” “PI3K/Akt pathway,” “JAK-STAT pathway,” “endothelin receptor antagonists,” “stem cell therapy,” “gene therapy,” or “endothelial biomarkers”).

Search syntax was adapted for each database to ensure comprehensive coverage of key topics, including EndMT, endothelial cell proliferation/apoptosis, and endothelial-targeted interventions. Original research papers, review articles, and clinical studies were considered. Studies unrelated to PAH or not involving endothelial mechanisms were excluded. Two independent investigators conducted duplicate removal (using EndNote software), title/abstract screening, and full-text assessment. Discrepancies were resolved through discussion with a third reviewer until consensus was reached. In total, 66 studies were included, forming the evidence base for this mini review.

## 3 EndMT and PAH

EndMT is a process in which ECs transform into mesenchymal cells (Yun et al., 2020). During EndMT, ECs lose endothelial markers such as vascular endothelial cadherin (VE-cadherin) and platelet endothelial cell adhesion molecule-1 (CD31), while gradually acquiring mesenchymal markers, including α-smooth muscle actin (α-SMA), fibronectin, and vimentin. This transition disrupts cell-cell interactions, causing ECs to detach from the vascular endothelial monolayer, lose polarity, and undergo cytoskeletal remodeling. As a result, ECs exhibit significant morphological, functional, and phenotypic changes, gaining enhanced migratory and invasive properties. EndMT plays a crucial role in pathological processes, contributing to tissue remodeling, increasing stromal cell populations, exacerbating fibrosis, and impairing tissue function.

In PAH, EndMT is a major contributor to pulmonary vascular remodeling (Gorelova et al., 2021). Monteiro et al. confirmed the presence of EndMT in PAH patients at the ultrastructural level (Monteiro et al., 2021). Similarly, Good et al. identified EndMT in a hypoxic PAH mouse model, reporting that 5% of PAECs co-expressed both endothelial and smooth muscle cell (SMC) markers (Good et al., 2015). Unlike genetically stable ECs under normal conditions, EndMT-derived cells acquire proliferative and migratory capacities (Alvandi and Bischoff, 2021). *In vitro* studies further demonstrate that EndMT cells promote mesenchymal cell proliferation and enhance migratory capacity through paracrine signaling. Additionally, they stimulate angiogenesis and upregulate proinflammatory cytokines, including interleukin-6 (IL-6), interleukin-8 (IL-8), and tumor necrosis factor-α (TNFα), which in turn recruit inflammatory cells to the endothelium, exacerbating disease progression.

Dysregulated bone morphogenetic protein (BMP) and transforming growth factor-β (TGF-β) signaling are key initiators of EndMT in PAH (Gaikwad et al., 2020). Mutations in BMP receptor II (BMPR II) have been identified in PAH patients and are associated with increased susceptibility to EndMT. These mutations impair BMP signaling, which normally inhibits EndMT and maintains endothelial stability. Consequently, the loss of BMP signaling leads to upregulation of TGF-β signaling, thereby promoting EndMT and vascular remodeling. Additionally, BMP-7 has been shown to suppress EndMT-driven proliferation and migration by inhibiting the mTORC1 signaling pathway (Yu et al., 2022). In PAECs, BMPR II and TGF-β receptor signaling induce growth arrest. However, the loss of these receptors results in increased expression of high mobility group A1 protein (HMGA1), which subsequently upregulates various transcription factors (Anbara et al., 2020). This cascade triggers extensive transcriptional reprogramming. Transcription factors, including Twist1, Snail, Slug, and hypoxia-inducible factors (HIFs, such as HIF-1α and HIF-2α), are integral regulators of EndMT (Zhang et al., 2025). In PAH patients, Twist1, Snail, Slug, and HIF-1α are both upregulated (Gallardo-Vara et al., 2023). Furthermore, PAECs from PAH patients exhibit elevated levels of HIF-2α (Zheng et al., 2022). These molecular alterations underscore the complex regulatory network governing EndMT and highlight its pivotal role in PAH pathogenesis.

## 4 EC proliferation, apoptosis and PAH

The structural and functional changes in PAECs are key driving factors in vascular remodeling and PAH (Evans et al., 2021). These alterations disrupt the balance between apoptosis and proliferation, thereby profoundly impacting vascular structure and function. In the early stages of PAH, widespread EC apoptosis leads to vascular injury and disruption of the endothelial barrier (Marinho et al., 2024). However, as the disease progresses, a subset of apoptosis-resistant ECs emerges within the pulmonary vasculature (Newcomb and Farkas, 2023). These cells exhibit enhanced survival, hyperproliferation, and pro-angiogenic characteristics. Selective pressures drive the expansion of these apoptosis-resistant EC subpopulations, allowing them to persist even in a hostile microenvironment (Zhang et al., 2024). These ECs disproportionately contribute to abnormal vascular proliferation and the formation of plexiform lesions, highlighting the critical role of endothelial heterogeneity in PAH progression (Mora et al., 2025). In addition, several studies have identified two distinct pulmonary capillary endothelial cell subtypes: general capillary (gCap) and alveolar aerocyte capillary (aCap) (Garcia-Hernandez et al., 2025). gCap cells function as progenitor-like endothelial cells that maintain capillary homeostasis and contribute to vascular repair, whereas aCap cells are primarily responsible for gas and solute exchange as well as mediating inflammatory responses (Dai, 2024). Dysregulation or reprogramming of these subtypes under pathological conditions may further exacerbate endothelial dysfunction and vascular remodeling in PAH. As endothelial dysfunction progresses, the vascular walls become stiffer and thicker, leading to impaired blood flow and contributing to PAH progression.

In PAH, the activation of multiple signaling pathways plays a critical role in endothelial dysfunction and pulmonary vascular remodeling. Key pathways, including TGF-β, Akt, Notch, JAK-STAT, Toll-like receptors (TLRs), vascular endothelial growth factor receptor (VEGFR), and NF-κB, regulate EC apoptosis and proliferation, thereby contributing to PAH progression.

### 4.1 TGF-β signaling pathway

The TGF family comprises a group of growth factors with diverse biological functions. These factors activate both Smad-dependent and Smad-independent signaling pathways, thereby regulating various cellular processes (Aashaq et al., 2022). Among them, TGF-β and BMP are the most critical members, playing a pivotal role in the pathogenesis of PAH, particularly in pulmonary vascular remodeling. In PAH, plexiform lesions exhibit microsatellite instability or loss of TGF-β receptor II expression in ECs (Wang et al., 2022). These abnormalities disrupt normal signal transduction, triggering pathological changes associated with PAH. Mutations in BMPRII lead to excessive EC proliferation, further exacerbating pulmonary vascular remodeling. Under hypoxic conditions, ECs with low BMPRII expression induce apoptosis by upregulating p53 and selectively promote the proliferation of anti-apoptotic ECs (Katseff et al., 2021). This selective proliferation is a key factor in the abnormal vascular remodeling characteristic of PAH. Although the incidence of PAH is lower in carriers of genetic mutations, PAH induced by the activation of the TGF-β signaling pathway may be linked to the interaction of EC heterogeneity, genetic modifications, and environmental factors. Further research is needed to elucidate the specific mechanisms.

### 4.2 Akt signaling pathway

Akt is a crucial regulator of cellular growth and plays a key role in various physiological processes, including cellular transcription, translation, disease pathogenesis, cell proliferation, angiogenesis, and EndMT (Chen et al., 2022). Activation of the PI3K/Akt pathway has been documented in coronary arteries, the aorta, and ECs, where it influences endothelial nitric oxide synthase (eNOS) activity, leading to NO production (Ding et al., 2021). Enhancing the proliferation, apoptosis, and angiogenesis of endothelial colony-forming cells (precursors of ECs) under hypoxia through the Akt/eNOS pathway may aid in the treatment of PAH (He et al., 2019). In PAH, endothelial dysfunction and cell proliferation are promoted by the activation of the PI3K/Akt/mTOR signaling pathway in ECs (Jin et al., 2022). In the monocrotaline (MCT)-induced PAH rat model, PAECs express phosphatase and tensin homolog (PTEN), upregulate PI3K, and phosphorylate Akt (Wang and Valdez-Jasso, 2021). Akt phosphorylation promotes EC proliferation and exacerbates PAH progression. *In vitro* studies show that hypoxia increases the expression of SIRT1 in PAECs, thereby activating the Akt signaling pathway. Akt activation enhances the stability of HIF-1α and increases the expression of the anti-apoptotic protein Bcl-2 (Yan et al., 2023). Through these mechanisms, the Akt pathway also supports the survival and expansion of apoptosis-resistant EC subpopulations, further contributing to vascular remodeling in PAH. Additionally, the presence of reactive oxygen species (ROS) amplifies the SIRT1/Akt signaling pathway, inhibiting EC apoptosis and promoting EC proliferation, further contributing to the pathological progression of PAH.

### 4.3 Notch signaling pathway

The Notch signaling pathway, which consists of Notch receptors, ligands, and intracellular effector molecules, plays a crucial role in regulating the expression of downstream target genes (Zhou et al., 2022). This pathway is closely associated with the pathogenesis of cardiovascular and pulmonary vascular diseases, including PAH. Furthermore, it is essential for EC proliferation, differentiation, and apoptosis. Studies have shown that Notch signaling is expressed at elevated levels in the lung tissue of idiopathic PAH and hypoxic rats (Zhang et al., 2022). *In vitro* experiments demonstrate that Notch1 promotes the proliferation of human PAECs by downregulating the cell cycle inhibitor p21 (Sahoo et al., 2021). Additionally, Notch1 inhibits apoptosis by upregulating the anti-apoptotic proteins Bcl-2 and survivin. These findings suggest that Notch1 activation supports endothelial cell survival and proliferation, contributing to vascular remodeling in PAH. Studies in animal models have further revealed the role of Notch signaling. In the MCT-induced PAH mouse model, inhibition of the BMPRII-Smad-Notch3 signaling pathway leads to EC apoptosis and EndMT, initiating pulmonary vascular remodeling (Zhang et al., 2015).

### 4.4 JAK-STAT signaling pathway

The JAK-STAT signaling pathway plays a crucial role in various cellular processes, including cell proliferation, differentiation, apoptosis, and immune inflammation (Xin et al., 2020). It has garnered significant attention in the pathogenesis of PAH. In the MCT-induced PAH rat model, researchers found that STAT3 phosphorylation levels in PAECs were significantly increased, along with an upregulation of DNA synthesis and cell proliferation markers (Shafiq et al., 2021). This suggests that STAT3 activation promotes EC proliferation. Both *in vivo* and *in vitro* studies further confirmed that blocking STAT3 signaling effectively inhibits EC proliferation in hypoxia-induced PAH (Zhang et al., 2018). Sustained STAT3 activation also favors the survival and expansion of apoptosis-resistant EC subpopulations, thereby reinforcing anti-apoptotic mechanisms and vascular remodeling in PAH (Roger et al., 2021).

### 4.5 TLRs

Toll-like receptors (TLRs) are pattern recognition receptors in the innate immune system. They play key roles in various biological processes, including cell proliferation, division, migration, and drug resistance (Duan et al., 2022). Particularly, TLR3 plays a significant role in PAH pathogenesis by regulating EC apoptosis and promoting pulmonary vascular remodeling. TLR3 expression is reduced in lung tissues and ECs of PAH patients (Turton et al., 2020). In pulmonary arteries with intimal plexiform lesions, TLR3 expression is completely lost in ECs. In TLR3-deficient mice, hypoxia exposure results in more severe PAH, further highlighting the role of TLR3 in maintaining pulmonary vascular integrity (Bhagwani et al., 2023). *In vitro* studies have shown that TLR3 deficiency promotes EC apoptosis, suggesting that the loss of TLR3 may contribute to the pathological apoptosis observed in PAH. Therefore, therapies that restore TLR3 function or mimic its protective effects could alleviate EC apoptosis and vascular remodeling in PAH, offering potential targets for disease management.

### 4.6 VEGFR

Vascular endothelial growth factor (VEGF) is a key growth factor that specifically targets ECs by binding to its high-affinity receptor, VEGFR, which is primarily located on the surface of vascular and lymphatic ECs (Kaufm et al., 2021). This interaction promotes EC proliferation, migration, and angiogenesis. In the PAH rat model, the VEGFR inhibitor Su5416 rapidly induces EC apoptosis by blocking the VEGFR pathway, while selecting anti-apoptotic EC clones, leading to severe plexiform lesion formation (Deng et al., 2023). In addition, Ma et al. found that VEGFR3 expression was reduced in the lung tissues of PAH patients. *In vitro* experiments showed that knocking down the multifunctional adapter protein β-arrestin 1 (ARRB1) in ECs reduced the phosphorylation of VEGFR3, thereby inhibiting VEGF-C-induced EC proliferation, migration, and angiogenesis (Ma et al., 2019). The VEGFR pathway thus not only promotes EC proliferation but also facilitates the survival and expansion of apoptosis-resistant EC subpopulations, amplifying pathological vascular remodeling in PAH. In hypoxic mice, ARRB1 deficiency further impaired VEGFR3 signaling and exacerbated PAH progression.

### 4.7 The other signaling pathways

**FIGURE 1:**
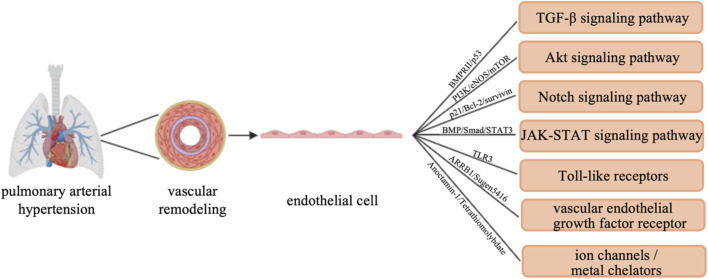
Signaling pathways targeting endothelial cells in PAH. In pulmonary arterial hypertension and pulmonary vascular remodeling, endothelial cells are involved in the following signaling pathways: TGF-β signaling pathway, Akt signaling pathway, Notch signaling pathway, JAK-STAT signaling pathway, Toll-like receptors, vascular endothelial growth factor receptor, and ion channels/metal chelators.

In addition to the VEGFR signaling pathway, various ion channels, metal chelators, and other factors also contribute to the progression of PAH (Hong et al., 2024). Anoctamin (Ano)-1, a calcium-activated chloride channel, plays a crucial role in regulating cell proliferation and the cell cycle. In PAECs from PAH patients, Ano-1 activation triggers the release of apoptosis-inducing factors, leading to cell apoptosis (Allawzi et al., 2018). This finding underscores the importance of ion channels in maintaining EC viability and highlights them as potential therapeutic targets for improving endothelial dysfunction in PAH. In addition, the role of metal chelators in the treatment of PAH has been increasingly recognized. Tetrathiomolybdate, a copper chelator, inhibits the proliferation of ECs isolated from lung tissue of PAH patients (Liang et al., 2021). This finding suggests that regulating metal ion levels and modulating EC behavior offers another potential therapeutic intervention for PAH. The interaction between metal ions and ion channels in ECs highlights the complexity of PAH pathophysiology and opens up possibilities for developing new therapeutic strategies (Figure 1).

## 5 Endothelial cell-secreted active substances and PAH

The pathogenesis of PAH is a complex process involving multiple factors and steps, characterized by the interaction between cells and their surrounding microenvironment (Adu-Amankwaah et al., 2025). This interaction is reflected not only in cellular energy metabolism and material exchange but also in signaling communication between cells, known as “crosstalk” (Su et al., 2025). In PAH, there is significant crosstalk between ECs, SMCs, and immune cells. ECs secrete active substances that influence the surrounding microenvironment and communicate with SMCs and immune cells, activating multiple signaling pathways.

### 5.1 Crosstalk with SMCs

The proliferation of SMCs is closely linked to the secretion of active substances by ECs (Cui et al., 2022). Zeng et al. found that anti-apoptotic ECs secrete microRNA-195-5p (miRNA-195), which promotes the proliferation and migration of pulmonary artery SMCs in PAH patients through the HIF-1α/miR-195/Smad7 pathway (Zeng et al., 2018). This mechanism illustrates how ECs regulate SMC behavior via specific molecular signals. Additionally, dysfunctional ECs secrete various factors, such as platelet-derived growth factor-β (PDGF-β), ET-1, and macrophage migration inhibitory factor (MIF), which induce the upregulation of forkhead box transcription factor M1 (FoxM1) in SMCs, thereby promoting SMC proliferation and pulmonary vascular remodeling (Dai et al., 2018).

Furthermore, PDGF secreted by ECs in PAH activates the PDGF receptor on SMCs, triggering the release of thromboxane and platelet-activating factor. This leads to platelet aggregation and the formation of *in situ* thrombosis. These processes not only exacerbate vascular remodeling but also increase pulmonary vascular resistance, further promoting the progression of PAH. Thus, ECs regulate SMC proliferation, migration, and vascular remodeling through multiple mechanisms, driving the pathological changes in PAH (Wu et al., 2020).

### 5.2 Inflammatory factors released by ECs

Immune inflammatory responses play a critical role in PAH-related vascular remodeling. Increased blood flow and pulsatility in PAH patients lead to PAEC damage, causing loss of barrier function and increased vascular permeability (Thenappan et al., 2018b). Compared to normal pulsating blood flow, high pulsating blood flow promotes the upregulation of TLR2 expression in ECs. TLR2 upregulation activates the NF-κB signaling pathway, further enhancing the expression of multiple proinflammatory molecules on the endothelial surface, including intercellular adhesion molecule-1 (ICAM-1), vascular cell adhesion molecule-1 (VCAM-1), E-selectin, and monocyte chemoattractant protein-1 (MCP-1). This leads to an aggravated inflammatory response in ECs, further promoting vascular remodeling and PAH (Oates et al., 2022).

Additionally, the classical signal transduction pathway of IL-6 is generally associated with anti-inflammatory function, while reverse signaling is linked to proinflammatory responses. Human vascular ECs express the IL-6 receptor (IL-6R) and glycoprotein 130 (gp130) on their surface, and their expression is regulated by proinflammatory stimuli (Xu et al., 2023). Reverse signaling through IL-6 enhances EC secretion of MCP-1, which subsequently recruits monocytes and macrophages, initiating immune-inflammatory responses (Kong et al., 2022).

Moreover, other cytokines such as tumor necrosis factor-α (TNF-α) and interleukin-1β (IL-1β) are critical mediators in PAH progression. TNF-α can impair endothelial nitric oxide (NO) production, induce endothelial apoptosis, and promote adhesion molecule expression, thereby facilitating leukocyte adhesion and vascular inflammation (Hurst et al., 2017). IL-1β further amplifies vascular inflammation through NF-κB and MAPK signaling, leading to increased secretion of IL-6, MCP-1, and endothelin-1, which collectively contribute to endothelial dysfunction and vascular remodeling in PAH (Agrawal et al., 2023). These studies highlight the crucial role of immune-inflammatory responses in endothelial dysfunction and vascular remodeling in PAH.

## 6 Diagnosis and treatment of PAH targeting ECs

In recent years, targeted therapies and diagnostic methods for ECs have become a research hotspot, providing new directions for early diagnosis, disease monitoring, and treatment of PAH. PAECs play a central role in the onset and progression of PAH. When EC dysfunction occurs, it often becomes a key factor in the development of the disease. ECs are essential for maintaining vascular homeostasis (Wang et al., 2025). They regulate physiological processes such as vasomotion, platelet aggregation, and inflammatory response by secreting bioactive molecules, such as NO, PGI2, and ET-1. However, in PAH, the disruption of EC function leads to EndMT, proliferation, migration, and EC-SMC crosstalk, which induces vascular remodeling, vasoconstriction, and impaired microvascular formation.

### 6.1 EC-targeted treatments

**FIGURE 2:**
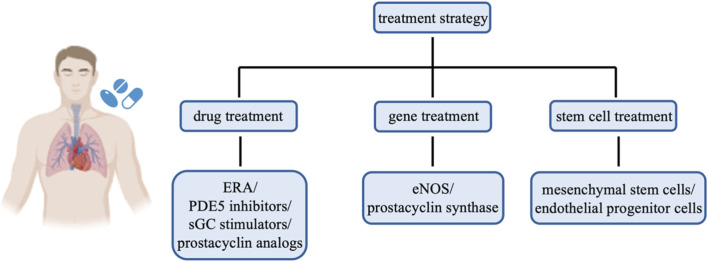
Strategies for PAH treatment. The treatment strategies for PAH mainly involve drug treatment, gene treatment, and stem cell treatment. Drug treatment includes ERA, PDE5 inhibitors, sGC stimulators, and prostacyclin analogs. Gene treatment includes eNOS and prostacyclin synthase. Stem cell treatment includes mesenchymal stem cells and endothelial progenitor cells.

Given the central role of ECs in the pathogenesis of PAH, recent studies have increasingly focused on therapeutic strategies targeting ECs. The aim is to alleviate or reverse the pathological process of PAH by restoring EC function, reducing EC damage, and regulating the interactions between ECs and SMCs. Current treatment strategies primarily include drug therapies, gene therapies, and stem cell therapies (Figure 2).

#### 6.1.1 Drug treatment

Currently, drug treatment remains the primary strategy for managing PAH, with drugs targeting EC function playing a crucial role in alleviating the condition. Endothelin receptor antagonists (ERAs) are among the most widely used targeted therapies (Jain and Isser, 2025). Endothelin, a potent vasoconstrictor secreted by ECs, causes pulmonary vasoconstriction. ERAs help alleviate this by inhibiting endothelin, thus reducing pulmonary artery pressure and alleviating PAH (Liu et al., 2023). Additionally, phosphodiesterase type 5 (PDE5) inhibitors, such as sildenafil, increase NO levels secreted by ECs, promoting vasodilation and improving blood flow, which in turn helps alleviate symptoms (Zhu et al., 2022).

Another important class of EC-targeted drugs is prostacyclin analogs, such as iloprost and epoprostenol, which enhance PGI_2_ signaling to promote pulmonary vasodilation and improve blood flow (Liu and Zhou, 2021). Moreover, research is exploring the potential of anti-inflammatory drugs and antioxidants in reducing EC inflammation and oxidative stress (Shen and Zhang, 2025). For instance, the antioxidant N-acetylcysteine (NAC) helps mitigate oxidative damage in ECs, thereby improving vascular function and contributing to PAH treatment (Vona et al., 2021).

Overall, drugs such as ERAs, PDE5 inhibitors, and prostacyclin analogs target EC function to alleviate vasoconstriction and promote vasodilation, making them the cornerstone of PAH treatment. Additionally, anti-inflammatory and antioxidant strategies are emerging as promising therapeutic approaches for improving EC function and reducing oxidative damage.

#### 6.1.2 Gene treatment

Gene therapy has emerged as a promising strategy to repair damaged PAEC function. By leveraging gene editing or gene transduction, protective genes can be introduced directly into ECs to restore normal function, reduce damage, and alleviate PAH. Recent studies have demonstrated that transfecting key enzyme genes, such as eNOS and prostacyclin synthase, enhances NO or PGI_2_ synthesis, effectively restoring EC function, improving vasodilation, and mitigating vascular remodeling (Shah et al., 2022; Zagrebelnaya et al., 2023). While gene therapy still faces challenges in clinical application, it presents a novel and promising direction for PAH treatment targeting endothelial cells.

#### 6.1.3 Stem cell treatment

Stem cell therapy has emerged as a promising treatment strategy for PAH, showing significant progress in preclinical and clinical research. By utilizing stem cells to repair damaged PAECs and promote EC proliferation, this approach aims to restore pulmonary vascular function, mitigate vascular remodeling, and reduce pulmonary hypertension. Among various stem cell types, mesenchymal stem cells (MSCs) have demonstrated great therapeutic potential, as they promote EC repair and regeneration, normalize EndMT, and reduce pulmonary vascular injury (Huang et al., 2020; Xu et al., 2022). In addition, both autologous and allogeneic stem cell transplantation, particularly using endothelial progenitor cells (EPCs), has been explored as a strategy to repair damaged ECs and restore endothelial barrier function (Zheng et al., 2023). Although still in the early stages of research, stem cell therapy holds promise for improving EC function, slowing vascular remodeling, and offering a novel therapeutic avenue for PAH treatment.

### 6.2 EC-targeting diagnosis

Advancements in targeted EC therapies have also driven innovations in PAH diagnosis. By monitoring EC function and assessing endothelial damage, new approaches are emerging for early diagnosis and disease progression monitoring. Traditional PAH diagnostic methods primarily rely on pulmonary artery pressure measurements and imaging techniques such as echocardiography, CT, and MRI scans (Rajagopal et al., 2024). While these methods assess pulmonary vascular morphology and function, they often detect abnormalities only at later disease stages. With the advancement of imaging technologies, researchers are exploring the use of EC-specific imaging probes to enable more precise pulmonary vascular assessments (Tomov et al., 2021). This approach has the potential to identify endothelial dysfunction at an earlier stage, facilitating timely intervention and guiding personalized treatment strategies.

In addition to imaging advancements, researchers are investigating blood-based biomarkers to assess EC damage in PAH. Potential biomarkers include EC-derived exosomes, microparticles, and specific endothelial markers such as VCAM-1 and E-selectin (Chen et al., 2024). Measuring these markers in the bloodstream may provide a non-invasive method to evaluate endothelial injury, allowing clinicians to detect PAH earlier and monitor disease progression more effectively.

## 7 Discussion

PAH is a life-threatening vascular disease characterized by pulmonary artery remodeling and increased pulmonary pressure, ultimately leading to right heart failure. Dysfunction of PAECs plays a central role in PAH pathogenesis. This review highlights the role of ECs in PAH, with a focus on EndMT, EC proliferation and apoptosis, key signaling pathways, and EC-SMC interactions.

Endothelial dysfunction in PAH extends beyond the abnormal occurrence of EndMT and is intricately linked to the dysregulation of key signaling pathways. Aberrant activation of pathways such as TGF-β, Akt, Notch, JAK-STAT, TLRs, and VEGFR plays a central role in regulating EC proliferation, apoptosis, and vascular remodeling. These pathways influence EC growth, migration, and survival, while also disrupting EC-SMC crosstalk. Consequently, EC dysfunction alters endothelial-smooth muscle signaling, contributing to structural remodeling of the pulmonary vasculature, increased vasoconstriction, and disease progression.

Current PAH treatments primarily focus on improving EC function, mitigating vascular remodeling, and reducing pulmonary artery pressure. Targeted EC therapies include drug therapy, gene therapy, and stem cell therapy. Among them, ERAs and PDE5 inhibitors are widely used in clinical practice, where they enhance EC function, alleviate vasoconstriction, promote vasodilation, and lower pulmonary artery pressure. Despite significant advancements in EC-targeted treatments and diagnostics, several challenges remain. Drug selectivity and safety pose major concerns, as many clinically available drugs show suboptimal efficacy and potential long-term side effects. Meanwhile, gene therapy and stem cell therapy, though promising, remain in preclinical stages, facing obstacles related to high costs, technical complexities, and regulatory hurdles before widespread clinical application. Additionally, large-scale clinical trials are needed to validate the therapeutic efficacy of EC-targeted interventions. Many potential drugs and treatment strategies are still under investigation, highlighting the need for continued research and innovation to optimize PAH management.

With the advancement of PAH research, early diagnosis and personalized treatment have become pivotal in improving patient outcomes. EC-specific blood markers such as VCAM-1 and E-selectin offer promising potential for early PAH detection, enabling clinicians to identify disease onset before significant symptoms appear. Additionally, imaging technologies are evolving, enhancing the assessment of pulmonary vascular function and structural changes. In the future, these biomarkers and imaging tools may facilitate early screening and risk stratification, identifying high-risk individuals before clinical symptoms manifest. Personalized treatment represents the future direction of PAH management. Given the heterogeneity of PAH pathogenesis, treatment strategies should be tailored to individual patient profiles. For instance, while some patients may exhibit EC dysfunction-driven PAH, others may primarily suffer from vascular remodeling due to excessive SMC proliferation. Precision medicine approaches, guided by molecular diagnostics, will enable clinicians to select targeted therapies best suited to a patient’s specific pathophysiological mechanisms, thereby enhancing treatment efficacy and minimizing adverse effects.

In conclusion, endothelial cell (EC)-targeted therapies hold significant promise for the treatment of pulmonary arterial hypertension (PAH). By gaining a deeper understanding of the role and mechanisms of ECs in PAH, we can uncover novel therapeutic targets, ultimately leading to better outcomes for patients. While current treatment strategies face certain limitations, ongoing advancements in research and technological innovation offer hope for more effective EC-targeted therapies in the future. The integration of early diagnosis, personalized treatment, and emerging therapeutic approaches will undoubtedly enhance the effectiveness of PAH treatments and significantly improve patients’ quality of life.
